# Supplementary material for: The Construction and Exploration of a Comprehensive MicroRNA Centered Regulatory Network in Foxtail Millet (Setaria italica L.)
Source: Front Plant Sci. 2022 May 6;13:848474. doi: 10.3389/fpls.2022.848474 (PMC9121102; doi:10.3389/fpls.2022.848474)
Supplement: Supplementary file 4 [file Data_Sheet_4.zip › Sit-miR5568_Seita.1G231300.1_514_TPlot.pdf]

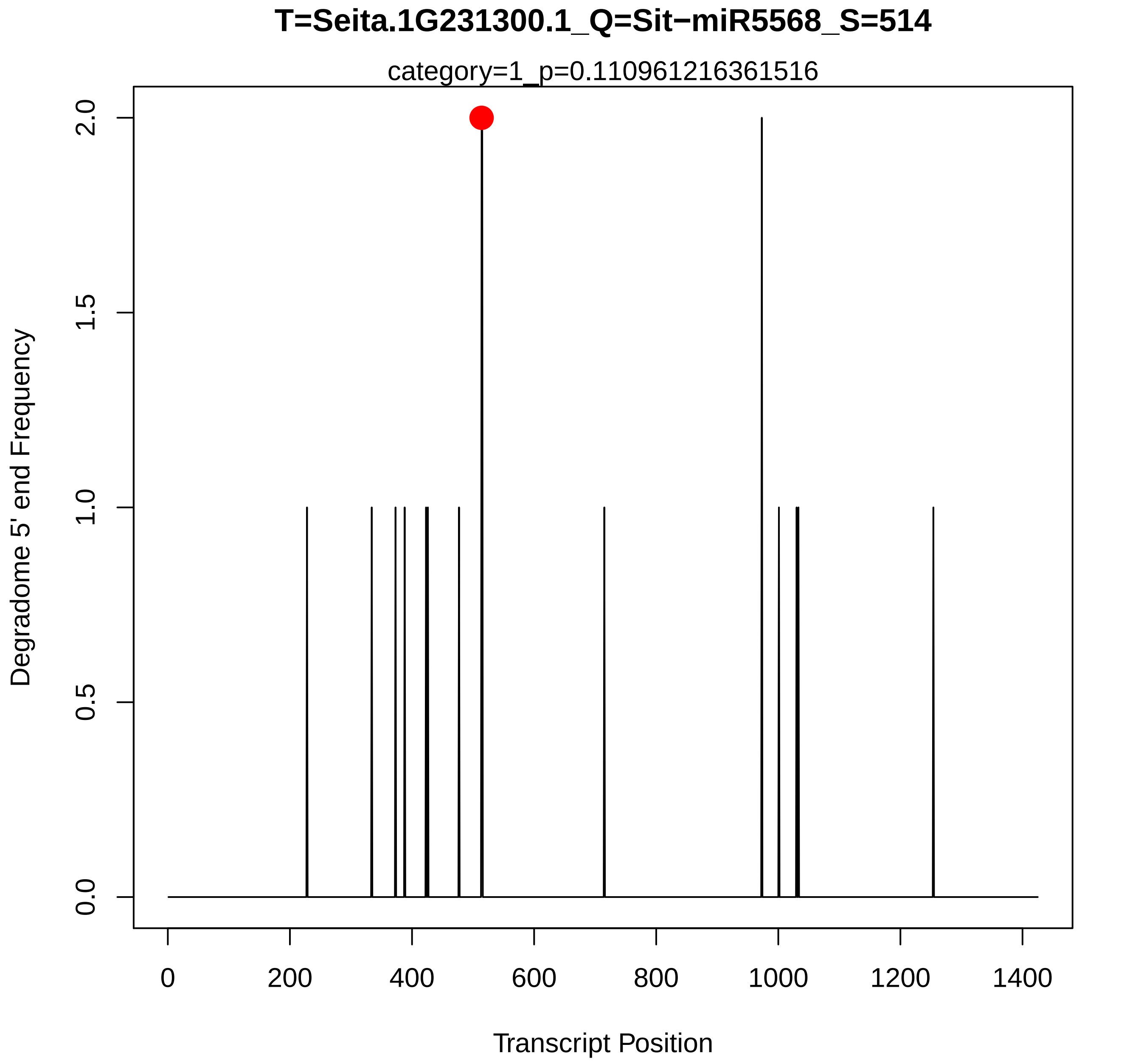

T=Seita.1G231300.1_Q=Sit−miR5568_S=514
category=1_p=0.110961216361516
Degradome 5' end Frequency
0.0
0.5
1.0
1.5
2.0
Transcript Position
0
200
400
600
800
1000
1200
1400
